# The pubertal development mode of Chinese girls with turner syndrome undergoing hormone replacement therapy

**DOI:** 10.1186/s12902-019-0403-2

**Published:** 2019-07-11

**Authors:** Song Guo, Jun Zhang, Yanhong Li, Huamei Ma, Qiuli Chen, Hongshan Chen, Minlian Du

**Affiliations:** Department of Pediatrics, The First Affiliated Hospital of Sun Yat-sen University, The 2nd Zhongshan Road, Number 58, Guangzhou city, Guangdong Province China

**Keywords:** Tanner stage, Hormone replacement therapy (HRT), Uterus, Turner syndrome, Breast

## Abstract

**Background:**

Further knowledge about the pubertal development mode of girls with Turner syndrome (TS) who have undergone hormone replacement therapy (HRT) is beneficial to the proposal of an optimal HRT regimen. This study examined the pubertal development mode of girls with TS who underwent HRT and evaluated the characteristics of optimal sex induction therapy in girls with TS.

**Method:**

We conducted a retrospective, longitudinal study over the past two decades at The First Affiliated Hospital, Sun Yat-sen University.

**Patients:**

Seventy-one patients with TS and two groups of normal Chinese girls.

**Results:**

The total investigation time was 3.00 (2.00, 4.66) years. The interval of each stage was significantly longer (*P* < 0.001) in the girls with TS than that in the normal Chinese girls, except for B2–3 (*P* = 0.011). The uterine volumes of the girls with TS in stages B2 and 3 were greater than those of the control group (*P* = 0.046), whereas the uterine volume of the control group was inversely greater than that of the TS group among those who reached stages B4 and 5 (*P* = 0.034). During HRT, the uterine volume grew significantly from all previous stages except for breast stage 5 (B_3 vs.2_: Z = − 2.031; *P* = 0.042; B_4 vs. 3_: Z = − 2.273; *P* = 0.023; B_5 vs. 4_: Z = − 1.368; *P* = 0.171). The paired data of 27 girls with TS showed that the uterine volume (17.93 ± 9.31 ml vs. 13.75 ± 6.67 ml) and width (2.54 ± 0.66 cm vs. 2.22 ± 0.36 cm) increased significantly during artificial cycles compared with before artificial cycles (t = − 2.79 and − 2.51, *P* = 0.01 and 0.018).

**Conclusion:**

HRT led to normal breast development in girls with TS; half of the girls with TS in our study reached Tanner stage B5, although the uterus ultimately developed suboptimally. The girls’ breasts and uteruses grew quickly at the beginning of HRT (stages B2–4). An optimal HRT regimen for girls with TS may specifically focus on Tanner stages B2–4 and artificial cycles.

## Background

Turner syndrome (TS) manifests as growth retardation, a lack of pubertal development, poor fertility [1] and a high miscarriage rate of 40–60% [2, 3]. Most girls with TS require hormone replacement therapy (HRT) to induce pubertal development, and mature uterine development allows better preparation for fertilization and a greater likelihood of successful gestation results [4]. However, the recent literature shows that for this population, achieving a mature uterine morphology is not a guarantee, even with HRT [5]. Moreover, some studies have investigated Chinese girls with TS [6], but to our knowledge, no existing study has examined Chinese girls with TS who were undergoing HRT. To investigate the pubertal development mode of girls with TS, ensure the integrity of the TS puberty database and describe the characteristics of optimal HRT, we conducted a longitudinal study that retrospectively examined 71 Chinese girls with TS who were undergoing HRT.

## Methods

This was a retrospective and longitudinal study conducted at a tertiary hospital (the First Affiliated Hospital of Sun Yat-sen University) in Guangzhou, China, between January 1998 and March 2018. The diagnosis of TS was made by G-band analysis of peripheral lymphocytes. The minimum number of lymphocytes for the diagnosis was 30. All the girls presented with no pubertal development or Tanner breast stage arrest. The control group subjects are separately discussed in the breast and uterine development sections.

### Subjects

The inclusion criteria were girls with TS who were undergoing HRT. The exclusion criteria included untreated hypothyroidism or hyperthyroidism and Y chromosomal material. Of the included patients, girls with TS who were assessed for breast development research were included based on the following criteria: 1. began pubertal development at no less than 12 years of age and 2. received HRT for at least 1 year. The two groups of control subjects were normally developing girls without a family history of precocious or delayed puberty.

### Methods

Girls with TS: Prior to 2007, estradiol valerate (Progynova®, Bayer Schering pharma) from 0.25 mg/d, qd, to 0.5 mg/d, qd, was initiated orally according to the doctor’s judgement, and the dose was increased every 3 to 6 months. An artificial cycle was induced until breakthrough bleeding occurred and began with a monthly dose of estradiolvalerat (1–4 mg/day for 21 days) together with medroxyprogesterone acetate (Provera®, Pfizer, New York, USA, 6–8 mg/day for 10 days). After 2007, therapy initiation followed the relevant guideline [7].

The participants’ anthropometric features and characteristics were measured, and the breast Tanner stages were assessed [8]. Two doctors performed the physical exam, and the results were accepted when the two doctors’ assessments were in agreement. When the doctors’ assessments differed, they were reassessed by a third doctor. A result consistent with one of the previous assessments was accepted; if the third doctor came to a new result, the subject was excluded. The initial age, bone age, karyotype, initial weight and body mass index (BMI), current estradiol valerate dose (the average dose was calculated if the dose changed during the recorded duration), serum follicle-stimulating hormone (FSH) and luteinizing hormone (LH) levels (Chemiluminescent microparticle immunoassay was used to measure the serum levels) before and after HRT, the total follow-up time, the time to each breast stage and the final stage (because a change of two breast stages could occur within one follow-up interval, the breast stage was recorded at the time or age at which any stage was reached, not the time at the beginning of the stage), and the addition of medroxyprogesterone. A transabdominal pelvic ultrasound examination was performed every 3–6 months. All of the ultrasound images were reviewed by an ultrasonologist at the same time. The uterine parameters included length, width, cross-section (anteroposterior diameter) and endometrium thickness. The uterine volume was calculated using the formula V = length × width × cross-section × 0.523 [9]. The maximum uterine volume was included in all of the presented uterine volume results.

The final breast stage was defined as reaching stage B5 or remaining at stage B3 or B4 for a median of at least 1.42 years or 1.92 years, respectively (the median intervals were 1.42 years for B3–4 and 1.92 years for B5; see Table 1). There were 39 girls who reached the final breast stage. The girls who reached the final breast stage were divided into the following two groups: the B5 group (*n* = 22) and the non-B5 group (*n* = 17). We analyzed the data to determine the possible effect of each variable on breast development.

**Table 1:** The interval time of each breast Tanner stage

Interval stage	N	Interval (M, P25, P75), years	N	Interval, years	Z	*P*
B2–3	21	0.75 (0.41,1.25)	139	1.06 (0.91, 1.00)	−2.51	0.01
B3–4	28	1.42 (1.00,2.08)	139	0.91 (0.91, 1.00)	−4.86	<0.001
B4–5	15	1.92 (1.41,2.84)	139	0.94 (0.91, 1.00)	−4.995	<0.001

Legend: We compared the duration data of each stage for girls with TS undergoing sex steroid HRT and normally developing Chinese girls during puberty and found that it in the girls with TS, breast stage 2 progressed more quickly, while breast Tanner stage 3 progressed more slowly

Footnote: The follow-up intervals of 3–6 months could include two changes in breast stage. We could not identify each change in stage; thus, we could not ensure that the number of patients during each interval remained the same

Control groups: We longitudinally followed the breast development of 139 normal Cantonese schoolgirls from baseline (Tanner stage B1) until they reached their final adult height (age 15.72 ± 0.84 years) and compared their breast development with that of the girls with TS. The 118 normally developing girls who served as controls for the uterine development research were cross-sectionally collected from girls aged 7 to 19 years who attended a wellness visit at our outpatient department from 2010 to 2015; the age, height, weight, breast stage, and transabdominal pelvic ultrasound examination results were recorded.

### Statistical analysis

GraphPad Prism version five (GraphPad Software, Inc.) and SPSS 20.0 were used for the statistical analyses. Frequencies and percentages were used for categorical variables, and the mean ± standard deviation (X ± SD) or median (M, P25, P75) was used for descriptive statistics. Student’s t test, nonparametric test, and the chi-square test were used to assess whether a given factor affected each breast stage interval or differed from that of a different uterine stage or time period. The factors associated with the final breast stage were calculated using logistic regression analysis. We used single-factor analysis to assess which factors might be associated with uterine volume development. A 2-tailed *p*-value less than 0.05 was considered significant.

## Results

The subjects were 71 patients with TS who received therapy at our institution [45, *X* (*n* = 39), 45, *X*/46, *XX* (*n* = 6) and structural abnormalities (*n* = 26, 9 with 45, XO/ 46, Xi (Xq); 7 with 46, Xi (Xq); 2 with 46, X, del (X) (qter - p11); 2 with 46, rX; 1 with 45, X, 1qh+;1 with 45, XO/ 46, Xi (Xq)/ 46, XX; 1 with 45, X,16 h-; 1 with 45, X, t (4; 5)(q31; q22)/ 46, XX, t (4; 5)(q31; q22); 1 with 46, XX/ 46, Xr (X)(p11; q22); and 1 with 45, XO/ 46, XXq23)]. Of those 71 patients, 63 were included for the assessment of breast development.

The 71 girls with TS began HRT at 15.71 ± 1.73 years of age, with a range from 11.5–19.75 years. The median age of the 63 patients with TS in the breast research project at the beginning of puberty induction was 15.82 (14.05, 17.59) years. The breast control group was composed of 139 girls aged 6.25 to 8.83 years (7.24 ± 0.38), and the median age at the entry into puberty (age at reaching B2) was 9.83 years (9.33, 10.33), with a range from 8.00–12.83 years. The uterine control group was composed of 118 girls with a median age of 9.81 years (8.25, 9.75) and a range from 7.00–19.00 years.

### Breast development

Table 1 shows the interval of each breast stage for the 63 patients with TS and 139 normally developing girls, who had BMIs of 18.79(17.68, 20.12) kg/m^2^ and 15.14(14.10, 16.56) kg/m^2^ (Z = 5.94, *P* < 0.001), respectively. The interval of each stage in the girls with TS was significantly longer than that of the normally developing Chinese girls. We did not find a significant difference between the monosomy TS patients (*n* = 22) and the patients with other karyotypes (*n* = 17) in the time to reach the final breast stage (2.479 ± 1.40 years vs. 2.988 ± 1.713 years, t = − 1.554, *P* = 0.298).

**Fig. 1:**
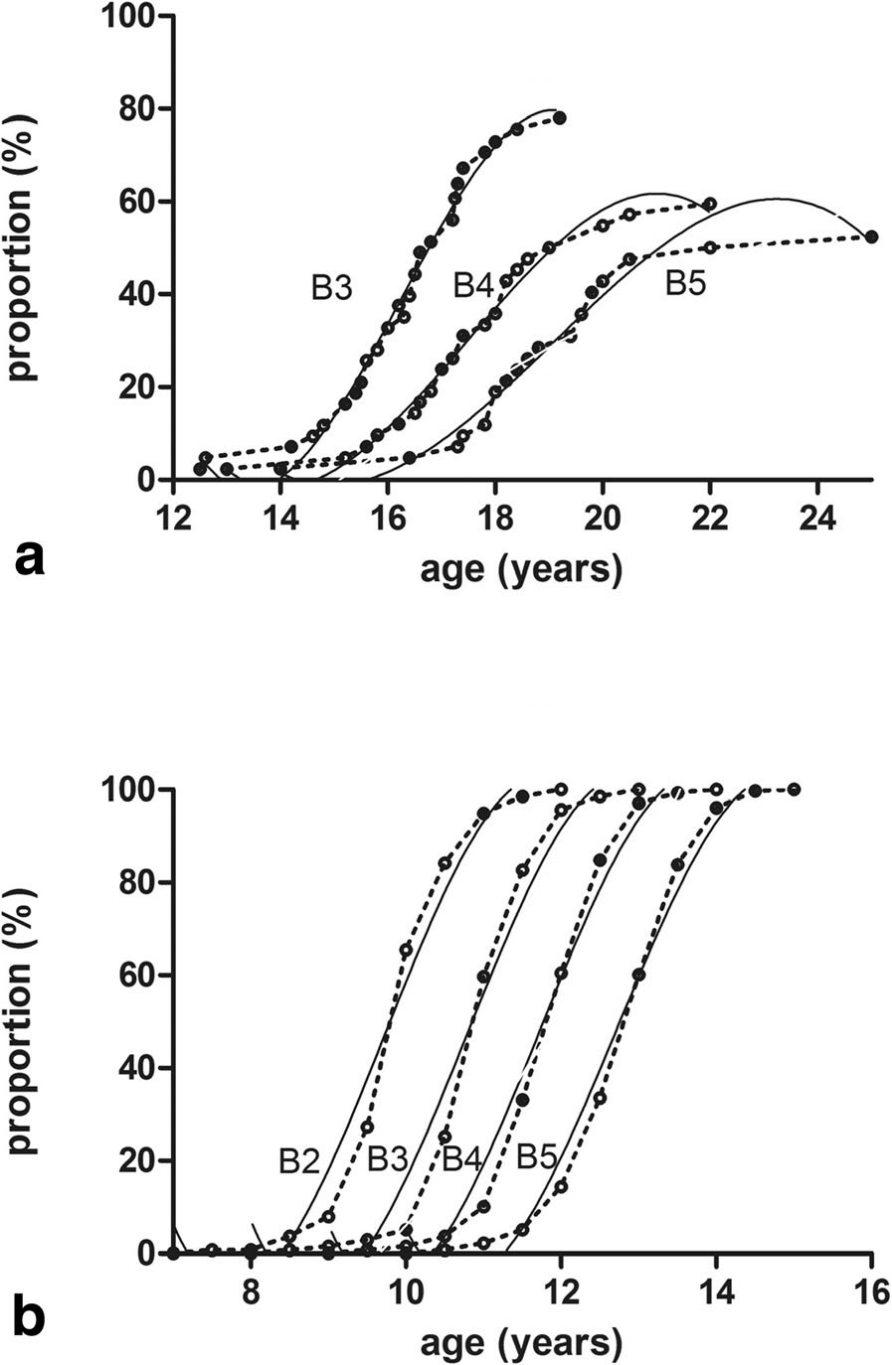
**a** and **b** Breast curves of 39 girls with TS who reached final breast stage and 139 normally developing girls. We computed the percentage of girls in each breast stage every 0.5 years starting at age 12 years and graphed them as the curves of age composition in breast stages B3 to B5 for girls with TS (the curve for girls with B2 was excluded because of the small sample size) (**a**) and normally developing Chinese girls (**b**)

The 39 girls who reached the final breast stage included 11 patients at stage B3, 6 at stage B4, and 22 at stage B5 (these patients reached stage B5 in 3.29 ± 1.46 years); over half of the girls reached stage B4 (71.79%) or B5 (56.41%). Figure 1 shows the breast development curves from our study. We divided the 39 patients into two groups [B5 (*N* = 22) and non-B5 (*N* = 17)] and analyzed factors that might correlate with whether the patient reached the Tanner B5 stage. We included the initial age, bone age, BMI, karyotype, addition of medroxyprogesterone, time to the final stage, total follow-up time, current estradiol valerate dose per kilogram, age at the addition of medroxyprogesterone, serum gonadotropin (FSH and LH) levels before and after therapy, and age when gonadotrop in was recorded before and after therapy as factors. The multivariate regression analysis indicated that the time to the final breast stage (3.29 ± 1.459 years vs. 2.02 ± 1.365 years, *P* = 0.006) and the LH level (14.876 ± 7.57 U/L vs. 21.95 ± 8.57 U/L, *P* = 0.01) before therapy were related to reaching breast stage B5 (OR = 2.493 and 0.876; 95%CI 1.132–5.493 and 0.779–0.985, respectively). Receiver operating characteristic (ROC) analysis showed that the 1/LH area under the curve (AUC) was 0.744 (cut-off ≥0.04); thus, the sensitivity and specificity of 1/LH were 90 and 50%, respectively. With the nonparametric test for the AUC and 0.5, the results showed a significant difference (*P* = 0.013).

### Uterine development

We analyzed the uterine development of the 71 girls with TS using breast Tanner stages. After HRT, the uterine volume, length, width, and cross-section grew significantly from the previous stage, except in the case of breast stage 5 (B2 vs. B3: Z = − 2.031, − 2.425, − 2.663, − 0.718; *P* = 0.042, 0.015, 0.008, 0.473; B4 vs. B3: Z = − 2.273, − 2.367, − 1.771, − 2.227; *P* = 0.023, 0.018, 0.077, 0.026; B5 vs. B4: Z = − 1.368, − 1.073, − 1.889, − 1.45; *P* = 0.171, 0.283, 0.059, 0.147; respectively, see Table 2). Interestingly, the uterine volume of the girls with TS in stages B2–4 grew faster than that of the girls in the control group; however, the growth inversely slowed when the patients reached stage B5.

**Table 2:** The characteristics of uterus parameters according to breast Tanner stages, M (P25, P75)

Stage		N	Duration since HRT (yr)	Estradiol valerate (mg/d)	Uterine volume (ml)		Uterine length (cm)		Uterine width (cm)		Uterine cross (cm)		Uterus endometrium (cm)
						Z, P		Z, P		Z, P		Z, P	
B1	TS	71	–	–	0.56 (0.35, 1.24)	−0.17, 0.089	1.80 (1.20, 2.40)	−0.792, 0.429	0.60 (0.50, 1.00)	−0.174, 0.081	1.00 (0.80, 1.10)	−2.86, 0.004	–
	control	18	–	–	0.89 (0.59, 1.50)		1.65 (1.56, 1.85)		0.80 (0.68, 1.10)		1.25 (1.00, 1.40)		–
B2	TS	33	0.33 (0.25, 0.42)	0.50 (0.28, 0.50)	2.86 (1.93, 6.37)*	−3.105,0.002	2.60 (2.20, 2.90)*	−4.04,<0.0001	1.25 (1.10, 1.50)*	−2.17, 0.03	1.80 (1.50, 1.70)	−2.27, 0.023	2.00 (1.00, 2.75)
	control	34	–	–	1.60 (0.95, 2.53)		1.90 (1.68, 2.20)		1.05 (0.90, 1.30)		1.55 (1.20, 1.90)		
B3	TS	56	0.83 (0.5, 1.23)	0.70 (0.50, 1.00)	5.22 (2.42, 11.00)^*^	−2.509, 0.012	2.85 (2.32, 3.68)^*^	−3.539,<0.0001	1.70 (1.33, 2.10)^***^	−1.802, 0.072	2.0 (1.50, 2.67)	−1.622, 0.105	2.00 (0.75, 3.00)
	control	48	–	–	2.77 (1.76, 4.26)		2.35 (2.00, 2.60)		1.35 (1.10, 1.80)		1.75 (1.40, 2.10)		
B4	TS	37	2.00 (0.75, 3.00)	1.25 (0.86, 2.00)	7.68 (4.82, 14.10)^**^	−1.999, 0.046	3.40 (3.00, 4.00)^**^	−0.959, 0.338	1.80 (1.60, 2.58)^*^	−2.214, 0.027	2.60 (1.90, 3.00)^**^	−1.868, 0.062	3.00 (2.00, 6.90)
	control	11	–	–	13.79 (9.27, 23.06)		3.70 (3.10, 4.20)		2.70 (2.20, 2.90)		3.10 (2.30, 3.90)		
B5	TS	17	3.58 (1.96, 4.71)	1.50 (1.00, 2.00)	11.69 (6.84, 13.65)	−2.124, 0.034	3.40 (3.05, 4.10)	−2.025, 0.043	2.10 (1.95, 2.63)	−1.869, 0.066	2.90 (2.15, 3.28)	−0.984, − 0.33	3.00 (1.00, 6.00)
	control	8	–	–	17.86 (14.44, 20.71)		4.40 (3.72, 4.80)		2.50 (2,33, 2.68)		3.05 (2.63, 3.80)		

Legend: We analyzed the uterine parameters and endometrium thickness according to breast Tanner stage and compared them with the previous stage. The uterine volume, length, width and cross-section increased significantly in every breast stage compared with the stage before except that in stage B5 (B2–3: Z = −2.031, −2.425, − 2.663; *P* = 0.042, 0.015, 0.008, 0.473; B3–4: Z = − 2.273, − 2.367, − 1.771, − 2.227; *P* = 0.023, 0.018, 0.077, 0.026; B4–5: Z = − 1.368, − 1.073, − 1.889, − 1.45; *P* = 0.171, 0.283, 0.059, 0.147; respectively). Compared with the normally developing girls, the girls with TS showed that uterine volume grew more quickly in stage B2 and B3 but more slowly in B4 and B5

Footnote: *: the data for the present stage compared with the last stage; *P* <0.05, **: *P* <0.01, ***P <0.001

A total of 42 girls presented breakthrough bleeding within 1.08 (0.50, 1.67) years post-HRT and began an artificial cycle; of these, 27 had full uterine parameter data available both of before and after the artificial cycle. The data showed that the uterine volume (17.93 ± 9.31 ml vs. 13.75 ± 6.67 ml) and width (2.54 ± 0.66 cm vs. 2.22 ± 0.36 cm) during the artificial cycle increased significantly compared with before artificial cycle induction (t = − 2.79 and − 2.51, *P* = 0.01 and 0.018), whereas the uterine length (4.85 ± 4.83 cm vs. 3.66 ± 0.65 cm, t = − 1.304, *P* = 0.204) and uterine cross-section (3.16 ± 0.69 cm vs. 2.99 ± 0.79 cm, t = − 1.022, *P* = 0.316) showed no significant changes.

To detect the possible factors influencing the induction of an optimal uterine development mode in girls with TS, we used single-factor analysis and found that the height before HRT, serum FSH and LH levels after HRT and the current dose and duration of estradiol valerate were positively related to the maximum uterine volume (*r* = 0.254, − 0.586, − 0.44, 0.499 and 0.364; *P* = 0.032, < 0.0001, 0.0005, < 0.0001, and 0.002, respectively; Table 3). We used single-factor analysis rather than multiple regression analyses to assess factors that might be associated with uterine development because no standardized dimension of an optimal uterus was available for grouping the girls with TS into optimal and suboptimal uterine groups. Therefore, further study on the cut-off value of the optimal uterine volume in girls with TS is essential.

**Table 3:** The correlation between characteristics and uterine volume, M (P25, P75)

Characteristic	N	M (P25, P75)	N	Uterine volume (max, ml)	R	*P*
Age (yrs)	71	15.75 (14.96, 16.92)	68	10.99 (5.91, 16.58)	−0.03	0.803
Bone age (yrs)	71	12.50 (12.00, 13.00)			0.154	0.216
Ht before HRT (cm)	71	144.8 0 (140.60, 147.95)			0.254	0.032^*^
Wt before HRT (kg)	71	38.50 (35.50, 42.75)			0.218	0.07
FSH age before HRT (yrs)	66	14.50 (13.00, 15.94)			0.134	0.199
FSH level before HRT (IU/L)	66	83.34 (59.65, 107.78)			−0.154	0.235
LH level before HRT (IU/L)	66	16.90 (12.17, 27.795)			−0.223	0.087
FSH level after HRT (IU/L)	42	44.34 (23.057, 61.88)			−0.586	< 0.0001^*^
LH level after HRT (IU/L)	42	13.82 (6.767, 16.445)			−0.44	0.005^*^
The present estradiol valerate dose (mg)	68	1.25 (1.00, 2.00)			0.499	< 0.0001^*^
The HRT time (yrs)	68	2.00 (1.27, 3.94)			0.364	0.002^*^

Legend: To detect the possible influencing factors for inducing an optimal uterine development mode in TS girls, we used single-factor analysis and found that the height before HRT, the serum FSH and LH level after HRT and the current dose and duration of estradiol valerate were positively related to the maximum uterine volume

## Discussion

The literature shows that up to 1/3 of girls with TS experience spontaneous puberty, but 90% of them experience puberty arrest or withdrawal and require HRT [10, 11]. In this paper, we presented the results of a retrospective observational and longitudinal study of 71 girls with TS who underwent HRT to interpret the pubertal mode; additionally, we tried to describe the characteristics of an optimal HRT regimen, which could support the need for more data to propose an optimal HRT regimen.

Piippo S et al. showed that all girls who were given percutaneous estradiol gel to induce puberty reached Tanner stage B4 after 5 years, and that 65% had reached stage B5 [12]. These proportions for Dutch girls were 87 and 50%, respectively [13]. Our study obtained similar results of 71.79 and 56.41%, respectively. Ankarberg-Lindgren C et al. found that older girls with TS showed a more sensitive response to estradiol valerate and that relatively small doses (0.08–0.12 μg/kg) could induce proper breast development [14]. However, even with the generally older patients in our study (the median age was older than 14 years), we did not find any significant correlations between the time to the final stage and the initial age, karyotype, estradiol valerate dose or the age at which medroxyprogesterone was given. Nevertheless, the time to the final stage, rather than the total follow-up time, was positively associated with the potential for reaching stage B5. A study by Doerr HG et al. of 188 girls with TS showed that most girls with monosomy and mosaic karyotypes reached stage B3 [15], although we did not find such differences in either the 39 girls who reached the final stage or the 22 girls who reached Tanner stage B5.

The documented progression of breast development differs according to the literature. The progression of breast development in girls with TS up to stage B5 is similar to that of normally developing Chinese girls. In contrast, the girls with TS showed significantly longer intervals for each stage except the B2–3 interval. This may be characteristic of the breast development process in these girls, even those with a higher prepubertal BMI, which has been positively correlated with puberty progress in normal girls [16]. Therefore, the breast development of girls with TS may be independent of the prepubertal BMI. The study also showed that the age at which the normally developing girls reached stage B2 was 9.83 years (9.33, 10.33); and the age for the girls with TS was just 6 years later than the mean pubertal age in normal girls (see in Fig. 1). Bannink E M et al. found a similar result, although their data indicated a time point just 2 years later for girls with TS than for their peers [13]. The B4–5 interval for the girls with TS was much longer than it was for the normally developing girls in our study, which was consistent with the results of Bannink E M et al.

We found that the time to the final breast stage and the serum LH level before HRT were correlated with the potential to reach stage B5.The serum LH level before HRT may have a predictive value for the breast response to HRT in girls with TS; therefore, we analyzed the reciprocal inverse of the serum LH level before HRT (1/LH) and found that the cut-off level of 1/LH was 0.04, with a sensitivity and specificity of 90 and 50%, respectively, regardless of age. We suspect that the 1/LH value may be a sensitive index for the breast response to HRT.

The girls with TS had prepubertal uterine dimensions before HRT that were comparable to those of normal prepubertal girls [13]. We assessed uterine development by using the Tanner stages [17] to provide a detailed study of the uterine development mode of girls with TS who underwent HRT.

In the analysis based on the breast Tanner stage, the uterine parameters increased significantly in a stepwise manner until breast stage B4, but not at stage B5. This outcome probably occurred because most of the girls with TS in stage B4 and B5 had undergone an artificial cycle. This result indicates that if we choose to assess uterine development according to breast stage, the analysis will be meaningful up to breast stage B4. Most of the literature shows that the uterine development of girls with TS is comparable to that of normally developing girls, even if the result is suboptimal [5, 13, 17], and some studies show a consistently smaller uterus starting in the prepubertal state and persisting to the post-HRT state [18]. Interestingly, our study showed that the girls with TS had comparable uterine dimensions in the prepubertal state and greater growth of the uterus than that of normal girls at Tanner breast stages B2–4; however, when the breast development stage reached B5, the uterine growth could not maintain its velocity. We suspect that the potential deficit of uterine dysplasia results from an insufficient X chromosome dosage, which might limit the function of sex steroid hormones in the uterus and eventually lead to a suboptimal uterus in girls with TS, even with HRT. This finding provides some indications that optimal HRT should focus on breast Tanner stages B2–4.

A total of 42 girls began the artificial cycle. When the maximum uterine parameters before and after the addition of medroxyprogesterone were compared, we only found an increase in the uterine width, which contributed to the significant difference in the uterine volume. This finding may indicate that an artificial cycle leads to strengthening of the uterine smooth muscle cells more than to changes in the cell length or proliferation, and it is consistent with the phenomenon of constant uterus growth in normal girls after menarche [19].

## Conclusions

Generally, HRT led to normal pubertal development in girls with TS, half of whom reached stage B5 in our study, but the uterus eventually remains in a suboptimal state. Low serum LH levels before HRT were associated with an increased likelihood of breast development reaching stage B5. The breast and uterus grew quickly at the beginning of HRT (stages B2–4) and the artificial cycle. This result provides some indications that the characteristics of optimal HRT should focus on breast Tanner stages B2–4 and the artificial cycle. The limitation of this study was that it was a retrospective analysis, and limit patients were included. Further research is needed to identify the optimal HRT regimen.

## Data Availability

The datasets used and/or analyzed in the current study are available from the corresponding author on reasonable request.

## Abbreviations

AUC: Area under the curve
BMI: Body mass index
FSH: Follicle-stimulating hormone
GH: Growth hormone
HRT: Hormone replacement therapy
LH: Luteinizing hormone
ROC: Receiver operating characteristic
TS: Turner syndrome
